# Elaboration and Validation of a Nomogram Based on Axillary Ultrasound and Tumor Clinicopathological Features to Predict Axillary Lymph Node Metastasis in Patients With Breast Cancer

**DOI:** 10.3389/fonc.2022.845334

**Published:** 2022-05-16

**Authors:** Yubo Liu, Feng Ye, Yun Wang, Xueyi Zheng, Yini Huang, Jianhua Zhou

**Affiliations:** ^1^ Department of Ultrasound, Sun Yat-Sen University Cancer Center, State Key Laboratory of Oncology in South China, Collaborative Innovation Center for Cancer Medicine, Department of Ultrasound, Sun Yat-Sen University Cancer Center, Guangzhou, China; ^2^ Department of Breast Oncology, State Key Laboratory of Oncology in South China, Collaborative Innovation Center for Cancer Medicine, Department of Breast Oncology, Sun Yat-Sen University Cancer Center, Guangzhou, China

**Keywords:** breast cancer, axillary lymph node metastasis, ultrasound features, lymphovascular invasion, nomogram

## Abstract

**Background:**

This study aimed at constructing a nomogram to predict axillary lymph node metastasis (ALNM) based on axillary ultrasound and tumor clinicopathological features.

**Methods:**

A retrospective analysis of 281 patients with pathologically confirmed breast cancer was performed between January 2015 and March 2018. All patients were randomly divided into a training cohort (n = 197) and a validation cohort (n = 84). Univariate and multivariable logistic regression analyses were performed to identify the clinically important predictors of ALNM when developin1 g the nomogram. The area under the curve (AUC), calibration plots, and decision curve analysis (DCA) were used to assess the discrimination, calibration, and clinical utility of the nomogram.

**Results:**

In univariate and multivariate analyses, lymphovascular invasion (LVI), axillary lymph node (ALN) cortex thickness, and an obliterated ALN fatty hilum were identified as independent predictors and integrated to develop a nomogram for predicting ALNM. The nomogram showed favorable sensitivity for ALNM with AUCs of 0.87 (95% confidence interval (CI), 0.81–0.92) and 0.84 (95% CI, 0.73–0.92) in the training and validation cohorts, respectively. The calibration plots of the nomogram showed good agreement between the nomogram prediction and actual ALNM diagnosis (P > 0.05). Decision curve analysis (DCA) revealed the net benefit of the nomogram.

**Conclusions:**

This study developed a nomogram based on three daily available clinical parameters, with good accuracy and clinical utility, which may help the radiologist in decision-making for ultrasound-guided fine needle aspiration cytology/biopsy (US-FNAC/B) according to the nomogram score.

## Introduction

Breast cancer is the most common cancer among women worldwide and the second leading cause of cancer-related death (1). A preoperative assessment of the axillary lymph node state is an essential issue in treatment decision-making. In patients with a clinically negative axilla, traditional staging by axillary lymph node dissection (ALND) has been replaced by surgical sentinel lymph node biopsy (SLNB) since the end of the 90s, due to the complications and morbidities of ALND (2). However, although SLNB is a less invasive method than ALND, the intraoperative pathological examination of SLNs significantly prolongs the operation time and increases costs (3, 4). Moreover, even in patients with positive SLNs, 56%–71% have no metastatic non-sentinel lymph nodes (5).

Ultrasonography (US) of the axilla is the method of choice for the assessment of the axillary nodal status in all patients with highly suspicious lesions or with a known breast cancer (6). US imaging shows acceptable accuracy for differentiating between benign and malignant breast tumors, but the accuracy of identifying positive lymph nodes varies (7, 8). Compared to conventional ultrasound, ultrasound elastography provides additional qualitative and quantitative information on tissue stiffness. Increased tissue stiffness of the primary tumor is associated with axillary lymph node metastasis (ALNM) in patients with breast cancer (9). Elastography features, including the elasticity imaging score and virtual touch tissue imaging quantification (VTIQ), have been used to supplement conventional ultrasound and predict ALNM in patients with breast cancers (10).

Currently, a nomogram is considered a precise tool that includes various characteristics of the disease to reflect the contribution of predictive variables to the outcome visually and directly. Several nomograms have been developed to predict ALNM in patients with breast cancer (5, 11–14). However, almost all the available nomograms were developed based only on clinical and pathological data, lacking US features (5, 11, 12, 14), or small sample sizes without validation (13).

Therefore, the aim of this study was to determine the axillary ultrasound features and tumor clinicopathological variables correlating with ALNM and develop a nomogram based on daily available parameters to predict ALNM in patients with breast cancer.

## Materials and Methods

### Ethics Statement

The study received hospital ethics committee approval (No. B2021-190-01), and written informed consent was obtained from each patient prior to data collection. The study was conducted in accordance with the Declaration of Helsinki to protect personal data. Patients who had received surgery, neoadjuvant chemoradiotherapy, or hormonal therapy were excluded.

### Study Population

Two hundred eighty-one patients with pathologically confirmed breast cancer between January 2015 and March 2018 were finally enrolled and randomly divided into two datasets at a ratio of 7:3. Majority of patients underwent sentinel node biopsy. If SLNB was positive, a complete ALND was performed. In addition, several early cases received direct complete axillary node dissection because of strong clinical suspects and patient preferences. The definition of negativity of ALN is negative SLN or negative ALN after direct ALND. Clinicopathological variables including age, BMI, menopausal status, tumor size, tumor location, presence of multifocal disease, histological type, histological grade, LVI, estrogen receptor (ER) status, progesterone receptor (PR) status, androgen receptor (AR) status, and the Ki-67 proliferative index as well as the results of a fluorescence *in situ* hybridization (FISH)-based analysis of HER2 gene amplification were collected *via* an electric medical record system. Several ultrasound and shear wave elastography (SWE) parameters, including node cortical thickening, fatty hilum presence, and VTIQ, were collected *via* the imaging system. The primary endpoint of this study was ALNM.

### Statistical Analysis

All patients were randomly divided into a training cohort and validation cohort at a 7:3 ratio with the “caret” package of R software (version 3.4.1; https://www.r-project.org/). Continuous variables were compared between the two groups using two independent sample *t* test or Mann–Whitney U tests, as appropriate, and categorical variables were analyzed using the χ^2^ test or Fisher’s exact test, as appropriate. The nomogram was constructed using data from the training cohort as described below: first, a univariate logistic regression analysis was performed to assess the ability of every variable to predict ALNM. Then, variables that reached statistical significance in the univariate analysis were fitted in the multivariate logistic regression analysis. Notably, ER and PR statuses, which were quantified as two of the most important drivers of breast cancer development, progression, and metastasis, showed no significant relationship with ALNM in the univariate analysis, while Ki-67, an alternative marker of cell proliferation, also had no correlation with ALNM in the univariate analysis. Given their important roles in breast cancer, the ER status, PR status, and Ki-67 level were further analyzed using the multivariate model. The backward selection procedure with the Akaike information criterion (AIC) score was introduced for variable selection to determine the independent variables that strikingly contribute to the patient prognosis. Hazard ratios (HRs) are presented with corresponding 95% confidence intervals (CIs). Finally, these final variables were incorporated to develop the nomogram with the rms package in R software. The performance of the nomogram was evaluated by assessing discrimination and calibration in both the training cohort and the validation cohort. Calibration was assessed graphically by plotting the relationship between actual probabilities and predicted probabilities and tested using Hosmer goodness of fit. DCA was applied to assess the clinical application of the nomogram. All tests were two sided, and P < 0.05 was deemed significant. The analyses were performed with SPSS for Windows (version 24.0, SPSS Inc., Chicago, IL, USA) and R software version 3.5.1.

## Results

### Baseline Patient Characteristics

The sample comprised 281 patients with a median age of 48 years, and 60% were postmenopausal. The final pathological results for lymph nodes after surgery were negative in 185 (65.8%) patients and positive in 96 (34.2%) patients, while 43 (15.3%) had ≥3 positive ALNs. ALNM positivity was 35.5% and 31% in the training and validation cohorts, respectively. No significant differences in baseline characteristics were observed between the training and internal validation cohorts. The detailed characteristics of patients with breast cancer in the training and validation cohorts are listed in **Table 1**.

**Table 1:** Characteristics of patients with breast cancer in the training and validation cohorts.

Characteristics	Total cohort (N = 281)	Training cohort (N = 197)	Validation cohort (N = 84)	P* value
Age (y)	48(43–58)	48 (42–57)	50 (43–60)	0.322
BMI (kg/m^2^)				0.256
<25	208 (74%)	142 (72.1%)	66 (78.6%)	
≥25	73 (26%)	55 (27.9%)	18 (21.4%)	
Postmenopausal				0.392
No	113 (40.2%)	76 (38.6%)	37 (44%)	
Yes	168 (59.8%)	121 (61.4%)	47 (56%)	
Tumor size (mm)	16.5 (12–21)	17 (12–21)	16 (12–21)	0.805
Tumor orientation				0.220
Parallel	109 (38.8%)	81 (41.1%)	28 (33.3%)	
No-parallel	172 (61.2%)	116 (58.9%)	56 (66.7%)	
Multifocality				0.379
No	260 (92.5%)	185 (93.9%)	75 (89.3%)	
Yes	21 (7.5%)	12 (6.1%)	9 (10.7%)	
Histological grade				0.430
I	7 (2.5%)	6 (3.0%)	1 (1.2%)	
II	131 (46.6%)	87 (44.2%)	44 (52.4%)	
III	127 (45.2%)	91 (46.2%)	36 (42.9%)	
Missing	16 (5.7%)	13 (6.6%)	3 (3.6%)	
Histology				0.476
IDC	241 (85.8%)	171 (86.8)	70 (83.3%)	
ILC	31 (11.0%)	19 (9.6%)	12 (14.3%)	
Other	9 (3.2%)	7 (3.6%)	2 (2.4%)	
LVI				0.385
No	178 (63.3%)	128 (65.0%)	50 (59.5%)	
Yes	103 (36.7%)	69 (35.0%)	34 (40.5%)	
ER				0.976
Positive (≥1%)	73 (26%)	145 (73.6%)	62 (73.8%)	
Negative (<1%)	207 (73.6%)	51 (25.9%)	22 (26.2%)	
Missing	1 (0.4%)	1 (0.5%)	0	
PR				0.639
Positive (≥1%)	108 (38.4%)	116 (58.9%)	53 (63.1%)	
Negative (<1%)	169 (60.1%)	77 (39.1%)	31 (36.9%)	
Missing	4 (1.5%)	4 (2.0%)	0	
AR				0.863
Positive (≥10%)	47 (16.7%)	32 (16.2%)	15 (17.9%))	
Negative (<10%)	207 (73.7%)	145 (73.6%)	62 (73.8%	
Missing	27 (9.6%)	20 (10.2%)	7 (8.3%)	
HER-2 status				0.647
Positive	82 (29.2%)	59 (29.9%)	23 (27.4%)	
Negative	198 (70.5%)	137 (69.5%)	61 (72.6%)	
Missing	1 (0.3%)	1 (0.5%)	0	
Ki-67				0.641
Low (<20%)	82 (29.2%)	56 (28.4%)	26 (31%)	
High (≥20%)	197 (70.1%)	139 (70.6%)	58 (69%)	
Missing	2 (0.7%)	2 (1.0%)	0	
Number of LN removed	14.5 ± 10.5	14.4 ± 11.0	14.7 ± 9.2	0.859
LN cortex thickness				0.76
<3 mm	191 (68%)	135 (68.5%)	56 (66.7%)	
≥3 mm	90 (32%)	62 (31.5%)	28 (33.3%)	
LN fatty hilum				0.253
Preserved	252 (89.7%)	174 (88.3%)	78 (92.9%)	
Obliterated	29 (10.3%)	23 (11.7%)	6 (7.1%)	
VTIQ	5.48 (4.34–6.69)	5.52 (4.56–7.00)	5.38 (4.05–6.54)	0.189

BMI, body mass index; IDC, invasive ductal carcinoma; ILC, invasive lobular carcinoma; LVI, lymphovascular invasion; ER, estrogen receptor; PR, progesterone receptor; AR, androgen receptor; HER-2, HER2/neu; LN, lymph node; VTIQ, virtual touch tissue imaging quantification.

*Comparison between training and validation cohorts.

### Independent Predictors of ALNM

**Table 2** summarizes the results of univariate and multivariate logistic regression analyses for ALNM in the training cohort. In the univariate analysis, tumor size, tumor orientation, LVI, ALN cortex thickness (≥3 mm), obliterated ALN fatty hilum, and VTIQ were significantly associated with ALNM and subsequently were subjected to multivariate analysis. Finally, three factors with the lowest AIC values, including LVI (P < 0.001), ALN cortex thickness ≥3 mm (P < 0.001), and obliterated ALN fatty hilum (P = 0.05) were identified as independent prognostic factors for ALNM.

**Table 2:** Univariate and multivariate analyses of ALNM in the training cohort.

Characteristic	Univariate analysis			Multivariate analysis		
	HR	95% CI	P value	HR	95% CI	P value
LVI			<0.001			<0.001
No	Ref			Ref		
Yes	7.73	3.99–14.95		9.03	4.14–19.69	
ALN cortex thickness			<0.001			<0.001
<3 mm	Ref			Ref		
≥3 mm	8.03	4.08–15.80		5.84	2.40–14.24	
ALN fatty hilum			<0.001			0.05
Preserved	Ref			3.85	0.98–15.09	
Obliterated	11.46	3.71–35.34				
Tumor size (cm)	1.07	1.03–1.12	0.002			
Tumor orientation			0.02			
Parallel	Ref					
No-parallel	2.1	1.12–3.88				
VTIQ	1.27	1.07–1.50	0.005			

ALN, axillary lymph node; LVI, lymphovascular invasion; HR, hazard ratio; CI, confidence interval; VTIQ, Virtual Touch Tissue Imaging Quantification; Ref, reference.

### Nomogram Development and Validation

**Figure 1:**
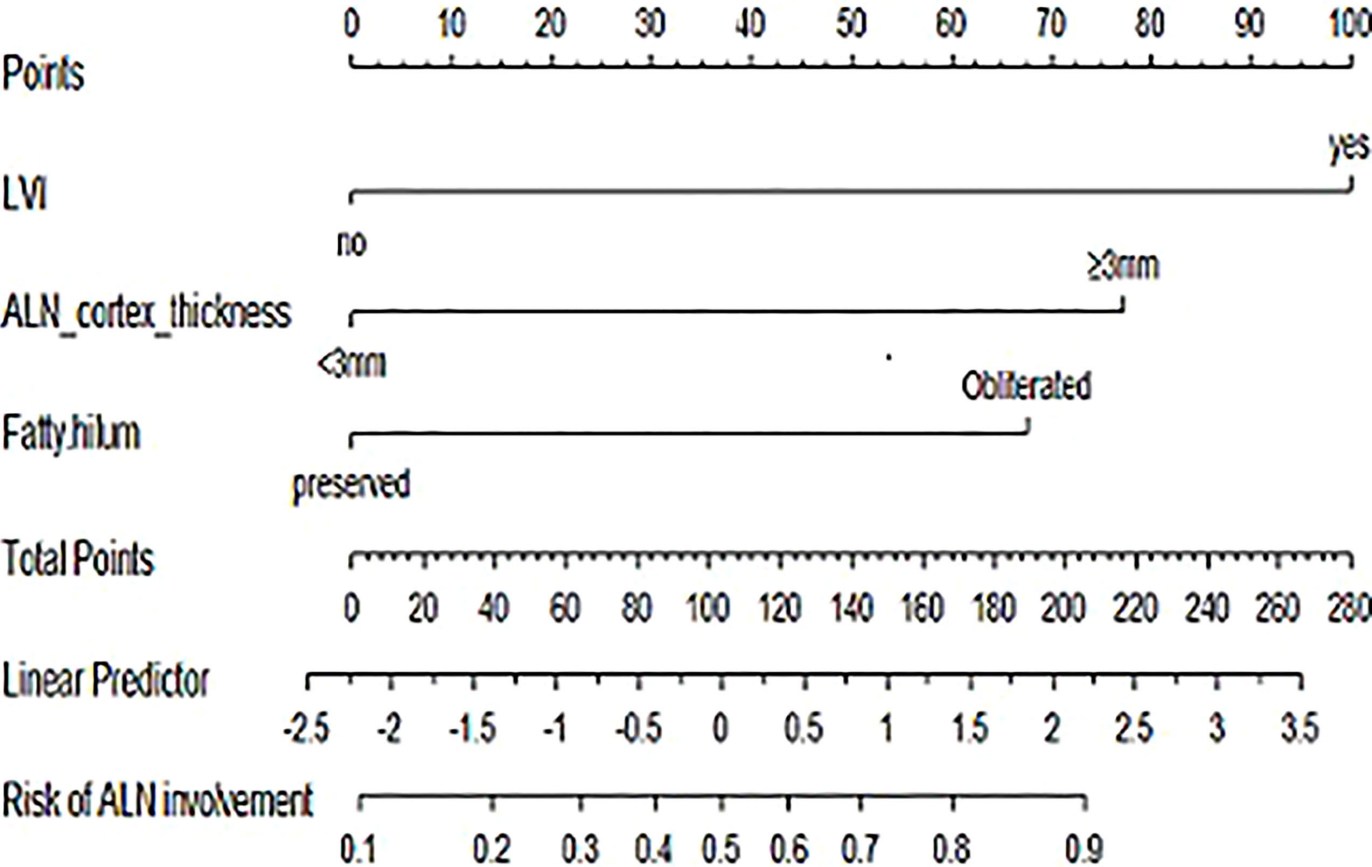
Nomogram for the prediction of ALNM in patients with breast cancer.

**Figure 2:**
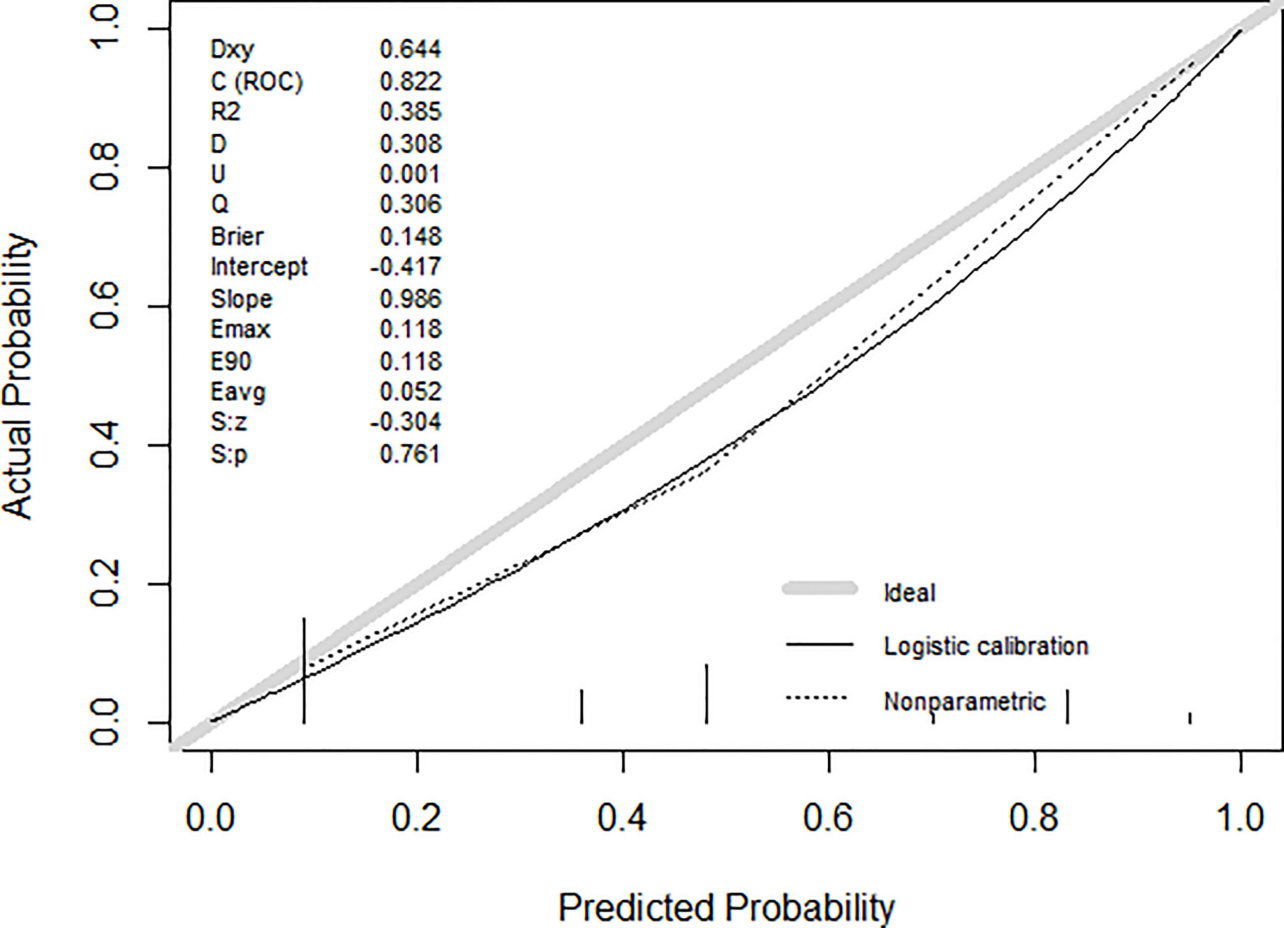
The calibration curves of the nomogram for the probability of ALNM.

**Figure 3:**
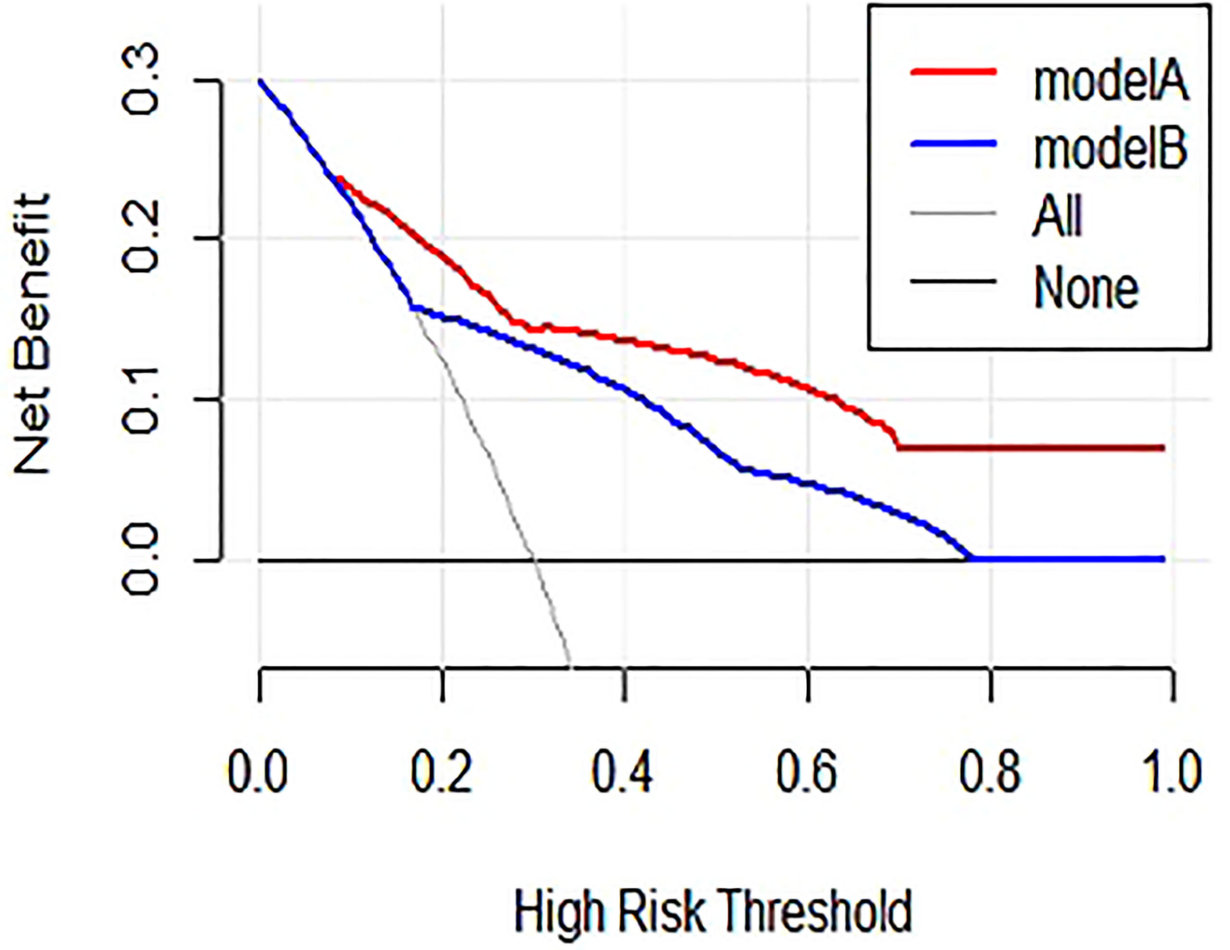
Results of decision curve analysis. Model A = combined model, model B = image-only model. Net benefit in relation to threshold probability for ALNM.

The nomogram for predictors of ALNM was developed based on the results of the multivariate analysis, as shown in **Figure 1**. The AUC of the model to discriminate ALNM is described in **Table 3**. The calibration curves revealed good agreement between the nomogram prediction and observation in both cohorts (**Figure 2**). DCA was used to evaluate the clinical utility of the nomogram. As shown in **Figure 3**, the nomogram showed great positive net benefits across wide ranges of ALNM risk in both cohorts, indicating its favorable clinical utility in predicting ALNM. Moreover, the combined model (model A) added more benefit in predicting ALNM than the image-only model (model B) at any set threshold probability.

**Table 3:** The AUC of the combined model and image-only model.

	Training cohort		Validation cohort	
	AUC	95% CI	AUC	95% CI
Predict ALNM				
Combined	0.87	0.81–0.92	0.84	0.73–0.92
Image-only	0.81	0.70–0.89	0.75	0.65–0.86

ALNM, axillary lymph node metastasis; AUC, area under the curve; CI, confidence interval.

Combined model (image-only model+ lymphovascular invasion).

Image-only model (ALN cortex thickness and obliterated ALN fatty hilum).

## Discussion

This study combined routine axillary US features and tumor clinicopathological characteristics to explore the predictors of ALNM. In the present study, three independent variables were identified in univariate and multivariate analyses: LVI, ALN cortex thickness, and obliterated ALN fatty hilum, which are readily available in daily clinical practice. The nomogram exhibited excellent predictive performance for ALNM, and its findings were also validated in the internal cohort, as indicated by the AUC, calibration, and DCA. According to our nomogram, if a patient achieves a score of 160 or higher, the probability of ALNM is >75%. This result should highly encourage the radiologist to perform a FNAC or FNAB on suspect lymph nodes, in order to obviate the need for SLNB during surgery or to address the patient to the more appropriate preoperative treatment. Because these three variables are routinely available even in basic hospitals or medically underdeveloped areas, this model may be widely applied.

Lymphovascular invasion (LVI) is a crucial step in the invasion-metastasis cascade that denotes the presence of tumor cells within lymphatic spaces, blood vessels, or both, at the peritumoral area and is identified morphologically by a microscopic examination of the primary tumor with or without endothelial-specific markers (15). Lauria et al. reported that LVI correlates with breast cancer lymph node metastases and a poor prognosis (16). A recent meta-analysis of 15 studies with 21,704 patients indicated that patients with early-stage breast cancer presenting LVI experience shorter overall survival, disease-free survival, breast cancer-specific survival, and more frequent local recurrence and distant metastases than those without LVI (17). In addition, LVI has been found to be the strongest independent predictor of ALNM (9, 18), which was also confirmed in the present study. As shown in the nomogram, LVI had the largest contribution to ALNM. As LVI can only be assessed using an invasive method and cannot always be evaluated in a fine needle aspiration biopsy, we compared the combined model and image-only model in the evaluation of ALNM. The performance of the combined model outperformed the image-only model (AUC of 0.84 vs. 0.75 for ALNM). In addition, DCA showed that the combined model provided a greater benefit in predicting ALNM than the image-only model at any set threshold probability, indicating the important role of LVI in the process of ALNM.

Ultrasonography is a non-invasive procedure used to evaluate metastatic disease and has good resolution for the detection of small nodes. The imaging characteristics of abnormal lymph nodes include cortical thickness ≥3 mm, prominent eccentric lobulation, and loss of the fatty hilum (19, 20). Here, two well-described features were used to define a lymph node as suspicious in our hospital: node cortical thickening (≥3 mm) and absence of the fatty hilum. The eccentric lobulation manifests focal cortical thickening, which is classified as abnormal cortical thickening in our institution. If either of the features was present, the lymph node was classified as suspicious (21, 22). Lymph nodal size was not evaluated in the present study, because enlarged lymph nodes can be caused by many non-cancerous causes such as bacterial, viral, or fungal infections. Some studies showed no relationship between nodal size and the presence of metastases (23, 24). In univariate and multivariate analyses, ALN cortex thickness (≥3 mm) and obliterated ALN fatty hilum were identified as significant predictors of ALNM, suggesting that ALN cortex thickness (≥3 mm) and obliterated ALN fatty hilum have a substantial predictive ability for ALNM.

Tumor size, tumor orientation, and VTIQ were associated with ALNM in the univariate analysis but not in the multivariate model. The ER status, PR status, and Ki-67 level, which are important drivers of breast cancer development, progression, and metastasis, showed no significant relationships with ALNM in the univariate analysis. Although the ER status, PR status, and Ki-67 level were further analyzed in multivariate analysis, no significant differences were identified. These variables were not significant in the multivariate analysis, possibly because of the powerful contribution of LVI to the ALNM model, as well as the characteristic change in node shape. In addition, collinearity and correlations of some of these factors among themselves may have played a role, along with the sample size.

Prior studies have reported several models for the prediction of ALNM. Dihge et al. (11) developed a prediction model that incorporated five parameters: age, mode of detection, tumor size, multifocality, and vascular invasion. The model showed an AUC of 0.74 for ALNM. Teixeira et al. (13) retrospectively analyzed the demographic, biochemical, and ultrasound characteristics of 74 patients. They reported that lymph node cortical thickness, presurgical tumor size, menopausal status, histological type, and tumor location were independent predictors of ALNM. A model consisting of these five variables was developed and produced an AUC of 0.848. Mittendorf et al. (5) constructed a nomogram based on eight variables: positive non-SLNs, number of SLNs identified, number of positive SLNs, SLN metastasis size, extranodal extension, tumor size, LVI, and histology, providing an AUC of 0.80 in the training cohort and 0.74 in the external cohort. Compared to these studies, our model only included three easily available parameters but showed comparable or better performance. For clinical application, the assessment of risk factors must be as convenient as possible. We propose that fewer variables indicate the better repeatability and operability of the model.

Additionally, an increasing number of studies have assessed the ALNM with the artificial intelligence (AI) technique. According to Zhou et al. (25), a deep learning algorithm-based approach performs better than experienced radiologists in the prediction of ALNM with an AUC of 0.89. Zheng et al. (26) reported that deep learning radiomics based on conventional ultrasound and shear wave elastography of breast tumor datasets shows an excellent discrimination of ALNM with an AUC of 0.902 in test cohorts. Guo et al. (27) developed a multicenter deep learning radiomics of the ultrasonography model to predict the risk of SLN and NSLN metastasis with the AUC of 0.86 in the training set and 0.81 in the test set. Sun et al. reported an AUC of 0.72 (SD ± 0.08) in predicting ALNM from US images using a deep learning technique in the test dataset (28). However, practical applications of AI are still being implemented in daily radiology practice (29). The limitations include lack of reproducibility, adaptivity, integration, and quality controls (30). Although the performance of our model is slightly worse than some models constructed using the AI technique, our model utilizes readily available clinical information, and the performance of the model including only three common factors is still good.

Some limitations should be acknowledged. First, this study was conducted at a single center, which is not fully representative of the entire population. However, our study was conducted in a tertiary medical hospital and included eligible patients treated in recent years without any other restriction. Second, some bias may inevitably exist due to its retrospective nature. However, the ultrasound data were acquired prospectively as part of our daily clinical practice and recorded before pathological results were known. Third, external validation in independent cohorts remains necessary before this nomogram is applied in other centers. However, the ultrasound examination of the patients in this study was performed by doctors with different levels of experience. All patients were randomly divided into a training cohort and a validation cohort.

## Conclusions

The present study developed a nomogram based on three routinely available parameters for predicting ALNM with good performance. Our nomogram may serve as an acceptable and adoptable clinical tool in the evaluation of ALNM, which may help radiologists to perform image-guided lymph node interventions. However, the model still requires further prospective study and external validation.

## Data Availability Statement

The original contributions presented in the study are included in the article. Further inquiries can be directed to the corresponding author.

## Ethics Statement

The studies involving human participants were reviewed and approved by the Ethics Committee of the Sun Yat-Sen University Cancer Center (No. B2021-190-01). The patients/participants provided their written informed consent to participate in this study.

## Author Contributions

YL, FY, and CSF drafted the manuscript. YL, YW, XZ, and YH participated in the ultrasonic examination and clinical data collection of patients. YL, FY performed the statistical analysis. FY, YW, XZ, and JZ helped in the verification of patients’ data and revised the manuscripts. JZ conceived of the study and participated in its design and coordination and helped to draft the manuscript. All authors contributed to the article and approved the submitted version.

## Conflict of Interest

The authors declare that the research was conducted in the absence of any commercial or financial relationships that could be construed as a potential conflict of interest.

## Publisher’s Note

All claims expressed in this article are solely those of the authors and do not necessarily represent those of their affiliated organizations, or those of the publisher, the editors and the reviewers. Any product that may be evaluated in this article, or claim that may be made by its manufacturer, is not guaranteed or endorsed by the publisher.

## Abbreviations

ALNM, axillary lymph node metastasis; ALN, axillary lymph node; US, ultrasonography; LVI, lymphovascular invasion; AUC, area under curve; CI, confidence interval; N, node; DCA, decision curve analyses; ALND, axillary lymph node dissection; SLNB, sentinel lymph node biopsy; LVI, lymphovascular invasion; VTIQ, Virtual Touch Tissue Imaging Quantification; BMI, body mass index; ER, estrogen receptor; ROC, receiver operating curves; AUC, area under the curve; HR, hazard ratios; IDC, invasive ductal carcinoma; ILC, invasive lobular carcinoma; AR, androgen receptor; HER-2, HER2/neu; LN, lymph node; Ref, reference.
